# Shaping ability of the profile 25/0.06 and protaper F2 in rotary motion, and reciproc in simulated canals

**DOI:** 10.7717/peerj.6109

**Published:** 2018-12-14

**Authors:** Gül Çelik, Murat Maden, Ahmet Savgat, Hikmet Orhan

**Affiliations:** 1 Faculty of Dentistry, Endodontics, Suleyman Demirel University, Isparta, Turkey; 2 Elmalı Dental Treatment and Prosthetic Center, Antalya, Turkey; 3 Faculty of Medicine, Biostatistics and Medical Informatics, Suleyman Demirel University, Isparta, Turkey

**Keywords:** ProFile, Reciproc, Single file, ProTaper, Shaping ability

## Abstract

**Background:**

Since the introduction of nickel–titanium (Ni–Ti) instruments to dentistry, a wide variety of Ni–Ti instruments have become commercially available. These Ni–Ti instruments are expensive, which limits their usage in developing countries and forces practitioners to use instruments repeatedly. Another problem is the possible prion cross-contamination associated with the multiple usage of endodontic instruments. In addition, the use of these instruments requires new skills and experience. In this article, the shaping capacities of two conventional rotary file systems, ProFile 25/0.06 and ProTaper F2, were reviewed and compared with the Reciproc single-file system.

**Methods:**

A total of 45 simulated canals with 40° curvature, in clear resin blocks, were prepared using conventional rotary systems consisting of ProFile orifice shaping (OS) #3 and final flaring #25/.06, Reciproc R25, and ProTaper shaping file SX and finishing file F2. Pre-and post-instrumentation images were analyzed at ten different levels, using AutoCAD 2007 software. The measurement positions were defined in 1-mm intervals: positions 0–3 established the apical part, positions 4–6 constituted the middle part, and positions 7–10 established the coronal part of the canal. The amount of removed resin, the transportation, instrumentation time, change in working length (WL), instrumentation fractures, and the presence of ledge were evaluated. Data were analyzed using ANOVA, Kruskal–Wallis and independent *t*-test (*p* < 0.001).

**Results:**

ProFile removed the least resin (*p* < 0.001) and caused less transportation than Reciproc and ProTaper, in total (*p* < 0.001). ProTaper caused more transportation ProFile and Reciproc in the apical part (*p* < 0.000). Reciproc caused more transportation than ProTaper and ProFile (*p* < 0.001), and the transportation tendency toward the inner aspect of the curvature in the middle part. Reciproc caused the less transportation than ProFile and ProTaper in the coronal part. The transportations tended to occur toward the outside of the curvature, except the middle part with Reciproc and at points 5 and 6 with ProTaper. There were no significant differences among the groups in terms of maintaining the original WL. Reciproc was significantly faster than the others group (*p* < 0.001). Only one instrument fracture (25/0.06 ProFile) was noted. All groups showed one ledge each.

**Discussion:**

The results of the present study showed that both ProFile 25/06 and ProTaper F2, combined with a file used for coronal enlargement (OS3 and SX), have the potential to create satisfactory canal shape in the curved root canals. Further studies using real human teeth are needed to confirm our results.

## Introduction

Dentist desires to complete the shaping of the root canal with a single file, even in the most difficult canal. Single-file systems have many advantages, such as time-consuming, the elimination of possible prion cross-contamination, a reduced file fatigue, and cost-effectiveness. The manufacturers continue to introduce new engine file systems to the market, to meet this demand of dentists.

An interesting development in canal shaping techniques using a single Ni-Ti file in a reciprocating motion to prepare curved canals in molar teeth was described ten years ago (Yared, 2008). In this technique, the canal is negotiated to the working length (WL) using a size 08 hand file. Then, the canal preparation is completed successfully with a ProTaper F2 instrument used in a reciprocating movement. Some studies reported satisfactory results using ProTaper F2 (Dentsply Maillefer, Ballaigues, Switzerland) in a reciprocating motion when cyclic fatigue resistance and shaping ability were measured (De-Deus et al., 2010; Kim et al., 2012; Kim et al., 2013). De-Deus et al. (2010) reported that the reciprocating movement extended the cyclic fatigue life of F2 ProTaper instruments when compared with the conventional rotary movement. Kim et al. (2012) confirmed the single-file technique using ProTaper F2 could be safely used under each reciprocating motion without creating an increased apical transportation in curved canals.

A nickel-titanium-based system, Reciproc (VDW, Munich, Germany), which shapes root canals using a single file, was introduced into the dentistry market several years ago. Reciproc has an S-shaped cross-section and sharp cutting edges that shape the canal by reciprocal back-and-forth motion in a 150° counter-clockwise and 30° clockwise rotation. The reciprocating instrument is first moved in a cutting direction, and then the instrument is reversed, to release. The file is made from a nickel-titanium-based m-wire subjected to a special heat treatment. The greatest feature of the M-wire nickel-titanium alloy is resistance to cyclic fatigue and greater instrument flexibility (Shen et al., 2006). The reciprocal movement is similar to the first application of balanced force introduced in 1985 by Roane, Sabala & Duncanson (1985), and it allows for use in automatic devices and even in severely curved root canals. The reciprocating working motion consists of a clockwise movement (release of the instrument) and a counter-clockwise movement (cutting direction). The counter-clockwise angle is greater than the clockwise angle. The manufacturer does not require the use of a glide path in the use of Reciproc files (Bürklein et al., 2012).

The ProFile, first introduced by Schilder in 1992, is the most studied canal instrument (Thompson & Dummer, 1997; Paqué, Zehnder & De-Deus, 2011), and is accepted as the gold standard (Lloyd, 2005). Jin et al. (2013) studied the performance of a reciprocating movement technique using only one ProFile 25/0.06 file for preparing curved canals, and suggested the reciprocating instrumentation technique using conventional Ni–Ti rotary file systems might have a comparable efficacy for the root canal shaping. Even though there is accumulating evidence of safety and shaping effectiveness of the Reciproc and ProTaper F2 when operated in a reciprocating motion, the knowledge of the shaping ability of ProFile 25/0.06 in rotary motion is lacking. Many studies investigating reciprocation with ProTaper F2 files and Reciproc have been reported. However, no studies comparing a single ProTaper F2 and a single #25/0.06 ProFile in rotary motion with the single system in reciprocating motion have been reported thus far.

Thus, the purpose of this study was to compare the shaping ability of ProTaper F2 file and ProFile 25/0.06 in rotary motion and R25 25/08 (Reciproc) in the curved root canal with reciprocation movement. The null hypothesis was that there was no difference between the techniques regarding any of the investigated outcomes.

## Materials & Methods

Forty-five simulated root canals in resin blocks (Lot # 1118484 Dentsply Maillefer) were subjected to operation in the study. The curvature of J-shaped simulated canals was 40 degrees and the length of the canals was 16.5 mm (Schneider, 1971).

The blocks were randomly divided into 3 groups (*n* = 15), and the following procedures were initiated. Groups PRF (ProFile orifice shaping (OS#3) and final flaring 25/.06 (Lot # 4259530 Dentsply Tulsa Dental, Tulsa, OK, USA)) and PRT (ProTaper shaping file SX and finishing file F2 (25/.05) (Lot # 1154441 Dentsply Maillefer)) were instrumented with continuous rotary movement with an electric motor (Technika; ATR, Pistoia, Italy) set at a speed of 300 rpm and torque of 30 (Technika motor setting value) in a 16:1 reduction handpiece. Group RR (Reciproc R25 (Lot #160072 VDW, Munich, Germany, Dentaire Company, la-Chaux-de-Fonds, Switzerland)) was instrumented with a reciprocating movement using a VDW Silver motor (VDW GmbH) following the manufacturer’s instructions. In the RR group, Reciproc files were used in reciprocating motion (150° counter-clockwise/30° clockwise, 300 rpm. The R25 instrument (Reciproc 25/0.08, VDW, Munich, Germany; lot number 160072) was introduced into the canal until resistance was felt and then activated in a reciprocating motion. The instrument was moved in the apical direction, by using an in-and-out pecking motion about 2–3 mm in amplitude, with light, apical pressure. After three pecking motions, the instrument was removed from the canal and cleaned with gauze soaked in alcohol. Light brushing movements were also applied against the canal walls when the file reached the WL. Final file size for Reciproc was #25/0.08.

The patency of the canals was confirmed using a #10 stainless steel K-file (Lot # 1185265 Dentsply Maillefer) and then the canals irrigated abundantly with saline. Each file was used in only one canal. The preparation time was noted by one of the authors. The preparation time also included the time needed to irrigate the canals, change the instruments, and check the apical patency.

### Assessment of canal preparation

Preparations in all groups were carried out by an author with 10 years of experience, and examination of canal morphology before and after instrumentation was performed by another author in a blinded manner based on the experimental groups. All prepared canals were assessed using composite pre- and post-instrumentation images. A camera (Canon EOS 500D DSLR, Tokyo, Japan) secured at a fixed distance (32 cm) from a microscope stage was used to capture the images, which were then saved on a desktop computer. To help align pre- and post- instrumentation photographs, each block was marked with 10 reference points. The composite images were analysed using image analysis software (AutoCAD, Autodesk, San Rafael, CA, USA).

AutoCAD was also used to assign a measurement scale and to compute the levels of the blocks removed at 20 positions (10 inner and 10 outer positions). The positions of measurement were defined using 1-mm intervals; positions 0 to 3 represented the apical part, points 4 to 6 constituted the middle part, and positions 7 to 10 represented the coronal plane part of the canal.

**Figure 1:**
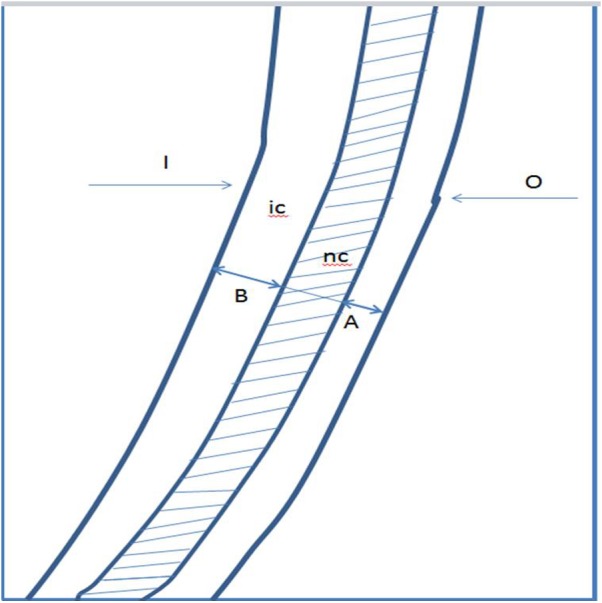
Drawing representing the composite image on which the measurements. I, inner side of the canal; O, outer side of the canal; nc, noninstrumented canal; ic, instrumented canal. (A) The distance between the outer limit of the noninstrumented canal and the outer limit of the instrumented canal. (B) The distance between the inner limit of the noninstrumented canal and the inner limit of the instrumented canal.

The distance between the outer limit of the noninstrumented canal and the outer limit of the instrumented canal (Fig. 1A), and between the inner limit of the noninstrumented canal and the inner limit of the instrumented canal (Fig. 1B) were measured. The following equations were used to determine transportation and the total amount of dentin removed:

\begin{eqnarray*}& & \text{Transportation}={|}A-B{|} (\text{A result of} `0\text{'} \text{indicates no transportation}) \end{eqnarray*}

\begin{eqnarray*}& & \text{Total amount of dentin removed}=A+B. \end{eqnarray*}

### Statistical analyses

Statistical analyses of data were carried out using SPSS 21 (IBM SPSS Inc., Chicago, IL, USA) software. Data were analysed using one-way multivariate analysis of variance (ANOVA) and least significant difference tests. The level of significance was set at 0.05.

## Results

Mean values and standard deviations of width values of canals shaped are shown in Table 1. ProFile was found to result in statistically significantly enlargement values overall and at each part and position of the canals (*p* < 0.001).

**Table 1:** Means (mm) and standard deviations at the different measurement positions and parts of the canal width after preparation with the different instruments.

Positions and parts	ProFile	Reciproc	ProTaper
1	0,18 ± 0,04^b^	0,24 ± 0,02^a^	0,25 ± 0,05^a^
2	0,22 ± 0,04^b^	0,30 ± 0,03^a^	0,31 ± 0,03^a^
3	0,24 ± 0,04^b^	0,33 ± 0,02^a^	0,33 ± 0,03^a^
4	0,64 ± 0,10^b^	0,87 ± 0,04^a^	0,89 ± 0,08^a^
5	0,27 ± 0,04^b^	0,39 ± 0,02^a^	0,38 ± 0,05^a^
6	0,31 ± 0,05^b^	0,44 ± 0,03^a^	0,40 ± 0,03^a^
7	0,37 ± 0,05^c^	0,48 ± 0,03^a^	0,45 ± 0,04^b^
8	0,92 ± 0,09^c^	1,31 ± 0,06^a^	1,23 ± 0,08^b^
9	0,40 ± 0,04^a^	0,48 ± 0,03^b^	0,50 ± 0,05^b^
10	0,41 ± 0,04^c^	0,48 ± 0,03^b^	0,54 ± 0,06^a^
Apical	0,64 ± 0,09^b^	0,87 ± 0,04^a^	0,89 ± 0,08^a^
Middle	1,21 ± 0,16^b^	1,71 ± 0,07^a^	1,67 ± 0,12^a^
Coronal	2,11 ± 0,20^b^	2,75 ± 0,11^a^	2,71 ± 0,12^a^
Total	3,14 ± 0,33^c^	3,96 ± 0,20^b^	4,47 ± 0,20^a^

**Notes.**

Values with different superscript letters were statistically different (*P* < 0.001).

The direction and amount of canal transportation (mm) at the different measurement points are shown in Table 2. In the apical part, ProTaper caused more transportation than ProFile and Reciproc and tended toward the outer aspect of the curvature (*p* < 0.000), whereas, Reciproc demonstrated greater transportation than ProFile and ProTaper and tended toward the inner aspect of the curvature, in the middle part (*p* < 0.000). In the coronal part, Reciproc showed less transportation than ProFile and ProTaper and tended toward the outer aspect of the curvature (*p* < 0.001). Overall, ProFile produced the less transportation than ProTaper and Reciproc and tended toward the outer aspect of the curvature (*p* < 0.004). There were significant differences in the amount of transportation among the file systems at all the measurement positions. At position 1, ProTaper generated more transportation than the other files (*p* < 0.000). At position 2, ProFile displayed the least transportation (*p* < 0.001). At positions 3, 7, and 8, Reciproc caused the least transportation among the files (*p* < 0.000), but at positions 4, 5, and 6, it caused greater transportation than the other files (*p* < 0.000) and tended toward the inner aspect of the curvature. No statistical difference was found among the groups, at positions 9 and 10.

**Table 2:** The direction and amount of canal transportation (mm) at the different measurement positions and parts.

Positions and parts	ProFile	Reciproc	ProTaper	*P* value
1	0,01 ± 0,03^b^	0,04 ± 0,04^b^	0,08 ± 0,07^a^	0,001
2	0,00 ± 0,03^c^	0,05 ± 0,04^b^	0,10 ± 0,05^a^	0,000
3	0,05 ± 0,04^b^	0,01 ± 0,03^c^	0,10 ± 0,06^a^	0,000
4	0,09 ± 0,05^b^	−0,10 ± 0,02^a^	0,02 ± 0,08^c^	0,000
5	0,07 ± 0,06^b^	−0,19 ± 0,11^a^	−0,11 ± 0,04^c^	0,000
6	0,05 ± 0,07^b^	−0,14 ± 0,10^a^	−0,10 ± 0,04^a^	0,000
7	0,08 ± 0,06^a^	−0,02 ± 0,04^b^	0,05 ± 0,05^a^	0,000
8	0,10 ± 0,05^a^	0,05 ± 0,03^b^	0,12 ± 0,058^a^	0,000
9	0,10 ± 0,05	0,09 ± 0,04	0,13 ± 0,08	0,244
10	0,10 ± 0,05	0,12 ± 0,06	0,10 ± 0,09	0,593
Apical	0,11 ± 0,04^b^	0,13 ± 0,06^b^	0,29 ± 0,14^a^	0,000
Middle	0,24 ± 0,09^a^	−0,48 ± 0,07^b^	0,27 ± 0,09^a^	0,000
Coronal	0,30 ± 0,12^a^	0,18 ± 0,0,6^b^	0,31 ± 0,11^a^	0,001
Total	0,74 ± 0,17^b^	0,91 ± 0,11^a^	0,97 ± 0,24^a^	0,004

**Notes.**

Values were calculated by subtracting the amount of resin removed at the inner side (concavity of the apical curvature) of the simulated canal from the amount of resin removed at the outer side (Negative value indicates the transportation tendency toward the inner aspect of the curvature). Values with different superscript letters were statistically different (*P* < 0.001).

There were no significant differences among the groups in terms of maintaining the original WL. The average WL loss in all groups was only 0.3 mm. Reciproc was significantly faster (23 s ± 4.4), followed by ProFile (52.8 s ± 3.7) and ProTaper (104.4s ± 16.5) (*p* < 0.001). Only one instrument fracture (25/0.06 ProFile) was noted. Canal irregularity was not observed in any group, and all groups showed one ledge each.

## Discussion

Since the introduction of Ni–Ti instruments to dentistry, a wide variety of Ni–Ti instruments have become commercially available. These Ni–Ti instruments are expensive, which limits their usage in developing countries and forces practitioners to use instruments repeatedly. This, however, poses problems from a standpoint of disease transmission (Scully, Smith & Bagg, 2003). In addition, the use of these instruments requires new skills and experience. In this article, the shaping capacities of two conventional files, ProFile 25/0.06 and ProTaper F2, operating in rotary motion, were reviewed and compared with the Reciproc single-file system.

Dentists are in general agreement that root canal shaping is the most time-consuming and exhausting part of root canal treatment. Manufacturers have been unable to introduce new equipment and tools to address this problem, changes have been made to cross-sections of root canal files, and new alloys are on the agenda. It was revolutionary for endodontists when manufacturers first marketed Ni-Ti and then motorized systems. Subsequently, the files used with systems started to show breakage problems, the more files that brake, the more difficult they are to remove, which increasing the number of instruments used and the consequent expense. Simultaneously, the reciprocating motion used in the Giromatic systems has started to regain popularity.

Attempts to shape root canals with a single file were first made with the ProTaper F2 in a reciprocating motion (Yared, 2008; De-Deus et al., 2010; Paqué, Zehnder & De-Deus, 2011; You et al., 2010). Subsequently, by utilising the advantages of m-wire alloys, single-file systems were marketed commercially. Some studies have been performed on the shaping capabilities of these instruments, and the results have been quite satisfactory (Kim et al., 2012; Kim et al., 2013; Bürklein et al., 2012). However, these instruments using a reciprocating motion, presented two drawbacks. First, the likelihood of instrument fracture in such cases is due to relative hardness resulting from the size, taper and cross-section of the instrument, rather than cyclic fatigue. Second, they make it necessary to create a glide path with additional hand files before using the F2 instrument in a reciprocating motion (Yared, 2008). It has been reported that 15 K file can be used safely with a reciprocating movement, while prevention of apical transportation, in the preparation a glide path (Kim et al., 2013).

M-wire Ni–Ti instruments are more flexible and resistant to cyclic fatigue than conventional Ni–Ti instruments (ProTaper F2 versus ProFile 25) (Johnson et al., 2008; De Almeida-Gomes et al., 2016; Ertaş, Çapar & Arslan, 2016). In clinical practice, these instruments are commonly associated with a risk of fracture. This risk may be reduced by performing coronal enlargement (Roland et al., 2002; Peters et al., 2003) and by creating a manual (Berutti et al., 2004) and mechanical (Berutti et al., 2009) glide path before using Ni–Ti rotary instrumentation. Since the cyclic fatigue resistance of the Profile and ProTaper files are lower than the Reciproc file, coronal enlargement was performed in both groups, in the present study.

The reciproc with #15 or #20 K-File glide path had shaping performance similar to those of other comparable file systems in the preparation of mandibular molars (Hwang et al., 2014; Marceliano-Alves et al., 2015; Çapar et al., 2014; Siqueira et al., 2013). Various glide paths created with the pathfiles were beneficial in creating minimal apical transportation, and reciprocating single-file systems were relatively centralized in the canal and with no difference between the pathfiles used in straight canals (De Carvalho et al., 2015).

Although glide path techniques have been proposed in single file systems, some studies have shown satisfactory results when these files are not used. The Reciproc without a glide path maintained the original canal curvature well and was safe to use (Bürklein et al., 2012; Bartols, Robra & Walther, 2017). The F2 without glide path preparation has created satisfactory root canal shaping as well as the full-sequence ProTaper system (Paqué, Zehnder & De-Deus, 2011). In the present study, we preferred coronal enlargement (using ProFile O.S 3 and SX) instead of glide path instruments.

A few studies have reported satisfactory cyclic fatigue resistance and shaping ability when using ProTaper F2 in reciprocating motion (De-Deus et al., 2010; Paqué, Zehnder & De-Deus, 2011; Kim et al., 2012). Based on these results, it is possible that canal shaping using a conventional rotary (Ni–Ti) file system in a reciprocating motion could be applied clinically. Hwang et al. (2014) stated that ProTaper F2 could not shape the canal to full length when it is used in a conventional continuous rotating motion. In comparison, ProFile is in more frequent contact with the dentin because of its larger radial fields, so the tip of the instrument may bind in the canal. In our study, ProTaper SX and ProFile 25 were used for coronal enlargement of the canal. Thus, ProTaper F2 and ProFile 25/0.06 have been used not only in the entire canal but also in the apical section.

The results of the present study show that statistically significantly less resin was removed at all measurement points in the ProFile group than in the other groups (*p* < 0.001). The amount of substance removed from root canals depends on the depth of penetration of the rotating instruments and the shapes of the instruments used. The convex triangular sections of ProTaper increase its cutting efficiency. Moreover, its taper ranges from 0.08 to 0.19, which is greater than that of ProFile and equal to that of Reciproc when used at the same level in the canal. However, the amount of substance removed was greater than the amounts reported in other studies (Paqué, Zehnder & De-Deus, 2011; Kim et al., 2013; Çapar et al., 2014; Kanagasingam et al., 2016). This could have been a result of increased duration of instrument use in the canal.

Canal transportation is defined as “Removal of canal wall structure on the outside curve in the apical half of the canal due to the tendency of files to restore themselves to their original linear shape during canal preparation; may lead to ledge formation and possible perforation” (American Association of Endodontists, 2003). Apical canal transportation is an undesirable deformity influenced by a wide variety of factors such as the type of instrument, size and type of alloy, instrumentation technique, and curvature. Insufficient cleaning is the main clinical result of the canal transportation. The original part of the canal remains untouched and unprepared so that insufficiently cleaned root canals are seen in cases of canal transportation (Hülsmann, Peters & Dummer, 2005). In addition, canal transportation may result over-reduction of sound dentin with the potential outcome of reduced fracture resistance and destruction of root integrity (i.e., an apical or strip perforation) (Schäfer & Dammaschke, 2006). It has been reported that apical sealing may be adversely affected if apical transport is greater than 0.3 mm (Wu, Fan & Wesselink, 2000). No transportation value recorded in the present study exceeded this limit.

Overall, the least transportation was formed by ProFile (*p* < 0.004). In the apical section, it was caused by the ProFile together with Reciproc (*p* < 0.004). The shaping ability of ProFile instruments has been investigated in a number of studies. In general, this system minimizes apical transportation by maintaining the original canal curvature well (Jardine & Gulabivala, 2000; Versümer, Hülsmann & Schäfers, 2002; Ayar & Love, 2004; Al-Sudani & Al-Shahrani, 2006). These positive features are believed to originate from the cross-section of the instrument, which is a radial area that allows the instrument to remain in the center of the canal by rotating 360 degrees (Koch & Brave, 2002). There has been no study reporting the performance of the ProFile alone or in combination with an additional auxiliary canal instruments such as pathfile or orifice shaper. In the middle part of the canal, most transportation belongs to the Reciproc group, and unlike the other groups, transportation is directed toward the inner part of the curvature. However, transportation did not exceed 0.03 mm at any point examined in this section. Overall, the transportations tend to occur toward the outside of the curvature, except the middle part with Reciproc and at positions 5 and 6, with ProTaper. When operated in a reciprocal motion, Kim et al. (2012) showed ProTaper F2 had a tendency of transportation toward the outer or lateral aspect of the curvature at 1- and 2-mm levels, and toward the furcation at 3- and 5-mm levels, respectively.

There are several methods to examine canal transportation, including radiographic imaging, cross-sectioning, computed tomography (CT), micro CT, cone beam CT (CBCT), and the use of simulated root canals (Unal et al., 2009; Paqué, Zehnder & De-Deus, 2011; Bürklein et al., 2012). Resin blocks were preferred in the present study, as they can simulate canals and are more successful in providing standardisation. Since no study examining the shaping ability of ProTaper F2 and Reciproc in resin blocks could be found in our comprehensive literature review, it is not possible to directly compare our results with others. However, a number of studies in which other investigative methods were used present data indicate that the F2 ProTaper transports at an acceptable level (Paqué, Zehnder & De-Deus, 2011; Çapar et al., 2014). In the present study, although the maximum apical transport was observed with the F2, this amount remained at the critical level as determined by Wu, Fan & Wesselink (2000). There are many reported studies on Reciproc, the first single-file system on the market. It has been shown that the shaping ability of this file, which usually completes shaping quickly, is satisfactory (Bürklein et al., 2012; Siqueira et al., 2013; Hwang et al., 2014; Çapar et al., 2014; Marceliano-Alves et al., 2015; Kanagasingam et al., 2016). It has been shown that the full-sequence ProTaper approach with Reciproc using CBCT straightened root canal curvatures created canal transportation comparable to those created by the other five files in the preparation of mesial canals of mandibular molars (Çapar et al., 2014).

In the present study, only one file was fractured in the ProFile group, while danger zone formation in one block and ledge formation in another block were recorded in the Reciproc group. Reciproc was significantly faster (23s ±4.4), followed by ProFile (52.8s ±3.7) and ProTaper (104.4s ± 16.5) (*p* < 0.000). In agreement with these findings, it has been reported by others that Reciproc instruments prepare canals significantly faster than other instruments.

According to the data provided in the present study, the ProFile was found to remove significantly less resin at all of the measurement positions and regions when compared with the Reciproc and ProTaper groups (*p* < 0.001). Moreover, the ProFile caused less transportation overall than all the other groups (*p* < 0.001). For this reason, the null hypothesis of the present study was rejected.

## Conclusions

The results of the present study showed that both ProFile 25/06 and ProTaper F2, combined with a file used for coronal enlargement, have the potential to create satisfactory canal shape in the curved root canals. However, the shaping ability of the ProTaper F2 and the ProFile 25/06 in rotary motion should be evaluated in S-Shaped canals and in premolars. This study being an in vitro analysis is limited as clinical scenario is different, hence further in vivo studies should be undertaken to substantiate the results of this study in vivo condition.

##  Supplemental Information

10.7717/peerj.6109/supp-1 Data S1 Raw data

## Additional Information and Declarations

### Data Availability

The following information was supplied regarding data availability: Raw data is provided in Data S1.
